# Quantifying the costs of interruption during diagnostic radiology interpretation using mobile eye-tracking glasses

**DOI:** 10.1117/1.JMI.5.3.031406

**Published:** 2018-03-02

**Authors:** Trafton Drew, Lauren H. Williams, Booth Aldred, Marta E. Heilbrun, Satoshi Minoshima

**Affiliations:** a University of Utah, Department of Psychology, Salt Lake City, Utah, United States; b University of Utah, Department of Radiology and Imaging Sciences, Salt Lake City, Utah, United States; c Austin Radiological Association, Austin, Texas, United States; d Emory University Hospital, Department of Radiology and Imaging Sciences, Atlanta, Georgia, United States

**Keywords:** interruption, diagnostic radiology, eye-tracking, visual search

## Abstract

What are the costs and consequences of interruptions during diagnostic radiology? The cognitive psychology literature suggests that interruptions lead to an array of negative consequences that could hurt patient outcomes and lead to lower patient throughput. Meanwhile, observational studies have both noted a strikingly high rate of interruptions and rising number of interruptions faced by radiologists. There is some observational evidence that more interruptions could lead to worse patient outcomes: Balint et al. (2014) found that the shifts with more telephone calls received in the reading room were associated with more discrepant calls. The purpose of the current study was to use an experimental manipulation to precisely quantify the costs of two different types of interruption: telephone interruption and an interpersonal interruption. We found that the first telephone interruption led to a significant increase in time spent on the case, but there was no effect on diagnostic accuracy. Eye-tracking revealed that interruptions strongly influenced where the radiologists looked: they tended to spend more time looking at dictation screens and less on medical images immediately after interruption. Our results demonstrate that while radiologists’ eye movements are reliably influenced by interruptions, the behavioral consequences were relatively mild, suggesting effective compensatory mechanisms.

## 1 Introduction

Interruptions are a common and potentially harmful occurrence in radiology reading rooms. Two recent workflow analyses found that radiologists are interrupted between once every 4^1^ to 12.1^2^ min during regular business hours. These interruptions are primarily in the form of medical questions during in-person or phone-call interactions. In fact, radiologists spend ∼37% of their time on call with nonimage-interpretive tasks.^1^ During after-hours radiology, interruptions may be even more common. At many academic institutions, after-hours phone calls are handled by a single radiology resident.^3^ A recent study found that on-call radiologists receive an average of 72 phone calls during a typical 12-h overnight shift.^4^

Despite the suggestive evidence that interruptions have negative consequences for radiologists, we know little about the risk associated with interruption in diagnostic radiology. Observational studies are a vital starting point, but do not provide a mechanistic account of what factors are critical in determining whether a given interruption or reading room environment will lead to adverse outcomes. Recent work has suggested that epochs of time where there are more phone calls are associated with more discrepancies between diagnoses among radiologists.^3^ However, it is not currently clear why more phone calls lead to more disagreements or how specific (or diffuse) these effects are with respect to specific phone calls. Indeed, Grundgeiger and Sanderson^5^ suggested that we currently lack evidence as to the extent interruptions lead to adverse events in healthcare due to “the descriptive rather than causal nature of most studies.” Meanwhile, although there is a basic science literature devoted to studying interruption, it is focused on specific tasks that may not apply to diagnostic radiology.

Interruptions have most often been studied in sequential computer tasks, where errors typically occur when the observer forgets the current step in the sequential task.^6^ An example is the UNRAVEL task, where observers are asked to complete a series of tasks categorizing two alphanumeric items based on seven sets of rules that must be completed sequentially. Interruptions in this literature are cognitively taxing tasks that are unrelated to the primary task (e.g., type a code into a pop-up window). Altmann and Trafton have developed a model^7^ to explain performance in the face of interruption. It successfully predicts performance when the interruption length is increased (which leads to more memory errors^8^) and the effects of practice (where more practice paradoxically leads to more errors immediately after interruption but better overall performance^9^). The UNRAVEL task is an invaluable tool for reducing complex tasks into component parts and investigating how these components interact with a variety of external factors, such as task difficulty. However, the task is very different from the task faced by radiologists on over-night call sessions. Most of the participants in the cognitive interruption literature are completing a task that they have never experienced before. In contrast, the current study examined the effect of interruptions on radiologists who were performing a task (evaluating a worklist of complex cases) that they do every day as part of their job. The extent to which the interruption literature applies to diagnostic radiology is therefore unclear.

The current work is the first study to experimentally manipulate whether specific cases were or were not interrupted. In experiment 1, there were four experimental cases that contained challenging findings. Each radiologist observer was interrupted during two of the four experimental cases. In experiment 2, there were two experimental cases. Thus, each radiologist was interrupted on one of the two cases. In designing studies with real medical cases, there is always a concern that it is very difficult to find a set of cases that are perfectly equated in terms of difficulty, complexity, type of case, and many other factors. Our procedure was designed to minimize these concerns by counterbalancing which case was interrupted between radiologists. Thus, case-level effects should average out across the population of observers when comparing interrupted and noninterrupted cases.

In clinical practice, radiologists may be interrupted during any part of their job. However, for current purposes, we focused on interruptions that occurred while they were evaluating complex CT cases. We specifically chose to focus on CT cases rather than two-dimensional (2-D) radiographs because of the high degree of variability in how long radiologists attend to radiographs. In order to ensure that we were able to initiate an interruption while the radiologist was in the midst of examining an experimental case, we chose to focus on challenging CT cases. In both experiments, interruptions took place ∼3  min after starting the experimental case. We reasoned that this was enough time that the radiologists would be unlikely to simply start the case over.

Based on previous work from our lab,^10^ we hypothesized that interruptions would lead to poor spatial memory for which areas had been examined prior to the interruption. This memory limitation could manifest in a number of different ways. One simple prediction is that poor memory for which regions had been previously examined would lead to a higher error rate. However, the cases chosen in experiment 1 contained just one or two abnormalities. It is therefore unlikely that the single interruption occurred at a moment that would ultimately cause one of these critical regions to be neglected. Moreover, in our prior work we found no evidence of an accuracy decrement in response to the interruption during a task modeled after a common radiology task. Importantly, the observers in this study were naïve observers performing a simplified lung nodule detection task rather than radiologists examining real cases. Despite these large differences, the goal of this basic research was to help inform the more applied work. Our basic science investigation of interruption cost in radiology-like tasks suggested that we are more likely to observe time costs than diagnostic accuracy costs. Moreover, our eye-tracking data suggested that the search was impaired in the period immediately after the interruption. In particular, we found that in the 30 s after interruption, observers were more likely to refixate on the same regions they had previously examined, and they were not good at resuming search in the same region that they had been investigating immediately prior to the interruption.^10^

In the current study, we employed a mobile eye-tracking system rather than the desktop system used in our prior research. This allowed us to use the same type of multimonitor setup that the radiologists use in actual practice while we monitored eye-position through unobtrusive glasses. However, the reduced spatial resolution of the mobile eye-tracking system and the fact that all images were not coregistered in the same system as the eye-tracker meant that we were not able to compute refixation rates. Instead, we coded fixation location in terms of broad areas of interest in order to assess what areas were receiving the most foveal attention before and after the interruption.

## 2 Methods

### 2.1 Demographic Information

Thirty-four radiologists participated in two experiments: 18 at the University of Utah (experiment 1) and 16 while attending RSNA (experiment 2). Participants in experiment 1 were recruited from the Radiology Department at the University of Utah School of Medicine. There was a mixture of residents, fellows, and attending physicians. We required that all participants in both experiments had experience with an overnight call situation where they were asked to monitor phone calls while working through a worklist of patients. This meant that first-year residents were not eligible for the study. As a result of the relatively unrestrictive participation requirements, there was a broad range of specialties across both experiments. Three of the radiologists did not fill out demographic information sheets. The remaining radiologists had graduated from medical school an average of 8 years prior to participation. Seven of the 15 radiologists were ABR certified and 8 were residents. Average age was 36 (range: 30 to 57, standard deviation: 7.3). They estimated viewing an average of 31 (range: 0 to 75, s.d.: 28.9) chest or abdominal CTs per week.

Participants in experiment 2 were recruited at the annual RSNA meeting. There was a mixture of residents, fellows, and attending physicians. We required that all participants had experience interpreting CT images. On average, radiologists had 9 years of experience with CT cases. Five of the 16 radiologists were ABR certified and 11 were residents. Average age was 43 (range: 30 to 65, s.d.: 13.3). They estimated viewing an average of 35 (range: 0 to 150. s.d.: 43.3) chest or abdominal CTs per week.

### 2.2 Experimental Design

The experiment took place at a modified workstation with two (RSNA) or four (Utah) monitors. In experiment 1, radiologists used one monitor for image and case navigation, two for medical image evaluation, and one for dictation. Experiment 2 used the same software for image display, but with two fewer monitors. In practice, this meant that one monitor was used to navigate between cases and evaluate medical images, while the other was used for dictation.

After reading and signing consent forms approved by the University of Utah Institutional Review Board, radiologists were shown a worklist of patients and told to read through the cases as quickly and accurately as possible. Images were displayed using Philips Isite software. The worklist was populated with a mixture of volumetric (e.g., chest CT) and 2-D (e.g., chest radiograph) images. Radiologists were asked to dictate their impressions of each case using Powerscribe dictation software. A radiologist familiar with the cases (author BA) coded diagnostic accuracy based on the participant’s dictation. Diagnostic accuracy was determined based on consensus judgment for critical findings according to our radiologist collaborators (BA and MH).

#### 2.2.1 Experiment One

**Fig. 1:**
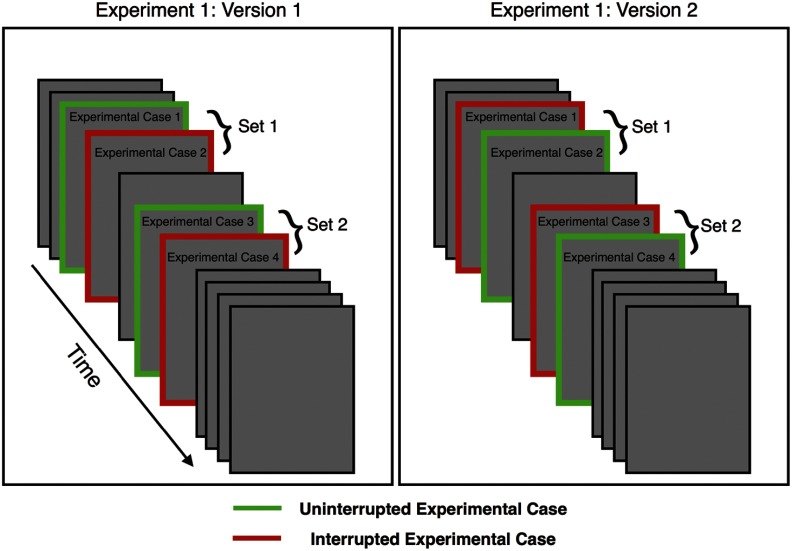
Experimental design for experiment 1. Each radiologist saw four experimental cases, two of which were interrupted by a telephone call. Experimental cases occurred in positions 3, 4, 6, and 7 for each radiologist. When quantifying the cost of interruption for each case, we compared performance on the paired uninterrupted case that fell immediately before or after the interrupted case. Identity of the interrupted cases was randomized across radiologists so that we could compare performance on the same case with and without interruption across radiologists.

In experiment 1, the worklist contained 11 cases. Participants were informed that they had 45 min to complete the worklist in order to ensure that they did not take an unrealistically long time on each case. There were four experimental cases in this experiment interspersed throughout the worklist (see Fig. 1). These cases were unique within the worklist in two ways:

1. In experiment 1, the experimental cases each contained at least one important finding that should have been identified in the case dictation. All other cases (nonexperimental cases, or filler cases) did not contain any pressing findings.
2. For each radiologist, half of the experimental cases were interrupted and the other experimental cases were not. The particular cases that were interrupted were counterbalanced across participants so that each case was interrupted an equal number of times across the experiment.

**Fig. 2:**
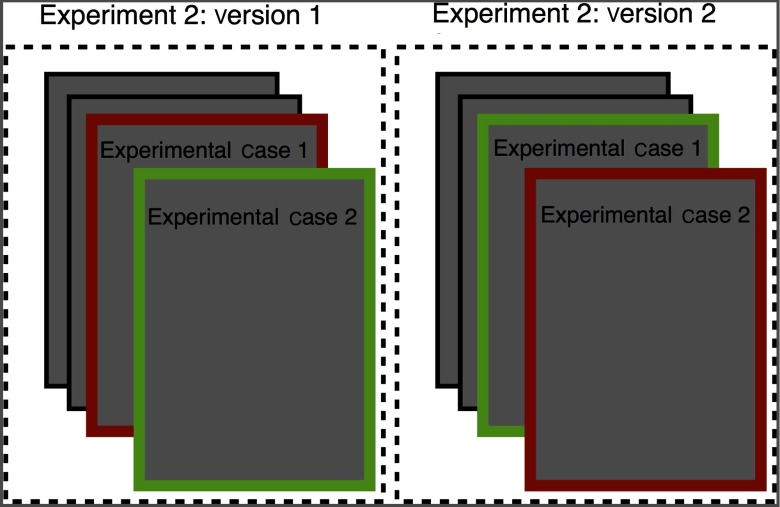
Experimental design for experiment 2. There were two experimental cases in experiment 2. Each radiologist was interrupted during one experimental case. Position of the experimental cases varied from positions 2 to 4. Identity of the interrupted case varied across radiologist so that performance on a given case could be compared with and without interruption across radiologists.

**Fig. 3:**
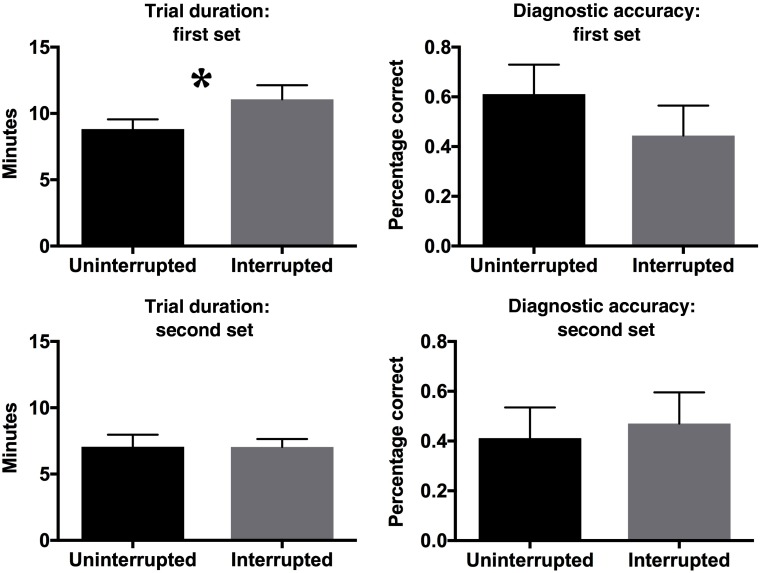
Diagnostic accuracy and trial duration for the first and second sets of trials in experiment 1. Error bars here and throughout the paper represent standard error of the mean.

The location of the four experimental cases within the worklist was pseudorandomized across radiologists. Case order was structured such that each radiologist saw two critical cases in position 3 and 4 (set 1), and two critical cases in position 6 and 7 (set 2), with one interruption in each set.

The seven additional filler cases were included to create a time-pressure for the participant and to discourage an expectation that a high proportion of the trials was to be interrupted by a phone call. These cases were chosen to be uncomplicated “normal” cases with no findings that necessitated follow-up (as judged by authors BA and MH). The cases were a mixture of radiographs, chest CT, and abdominal CT images. We did not further analyze the behavioral or eye-tracking results associated with these cases. Once the radiologist had read each case in the worklist, the experiment concluded.

Experiment 1 took place at a workstation in a radiology reading room during off hours. While explaining the experiment, the experimenter indicated that the phone next to the workstation might ring during the experiment and to treat the phone as they normally would while reading cases. Phone call interruptions took place on two out of four of the experimental cases. The interruption took place ∼3  min into each case. This time was chosen to ensure that the interruption did not take place near the end of the case and did not occur while the radiologist was first orienting to the case and determining what images to evaluate. Upon answering the phone, a prerecorded message simulating a clinician asked them to find a patient from a different worklist and provide a quick diagnostic interpretation. Both prerecorded messages asked the radiologist to examine the case of a patient who was not on the initial worklist who was complaining of abdominal pain. This meant that the radiologist had to exit the current worklist, open a second worklist, and find the patient in question. If the radiologist did not hear the name of the patient, the experimenter played the message again. Once the case had been found, the radiologist was asked to verbally indicate the diagnosis as if relaying it to the referring physician. Verbal responses were recorded using the audio input on the eye-tracking glasses. On average, radiologists devoted 2.2 (s.d.: 0.6) min to the telephone interruption before returning to the original case. We will return to limitations of this design in the discussion.

#### 2.2.2 Experiment Two

Radiologists read four cases in experiment 2 (see Fig. 2). There were two experimental cases, one of which was interrupted. A research assistant interrupted one experimental case to ask radiologists to stop what they were doing and fill out a form with demographic and radiological experience information. The short form contained nine questions and took an average of 1.6 (s.d.: 0.6) min to fill out. While filling out the paper form, the interrupted experimental case was still visible. We used eye-tracking to verify that the radiologists did not spend any time examining the case during the time we coded as during the interruption. As in experiment 1, the order of cases and which of the two cases was interrupted was randomized across radiologists.

### 2.3 Case Selection: Experiment 1

All cases were selected from a list of known “real-world” misses collected at the University of Utah for training purposes. Cases were selected after extensive review by two radiologists (BA and MH). The goal was to select cases that were neither too subtle nor esoteric, but were challenging. Although all four cases were missed in practice, both BA and MH agreed that they were clear in retrospect and might not have been missed. Neither case history nor previous images were provided with any of the cases.

Sternal fracture case: This was a chest CT case. Sternal fractures are a commonly missed finding.^11^ This was an unambiguous finding on lateral views of the sternum (see Fig. 5).
Bone case: This was an abdominal CT case with biopsy-proven metastatic disease in the spine that was unambiguous on sagittal imagining. The case also contained evidence of ulcerative colitis.
Kidney case: This was an abdominal CT case with acute diverticulitis and subtle pyelonephritis.
Pelvis case: This abdominal CT case included evidence of simple myositis/edema. The exact cause or underlying pathology is not known in this case. It was a nonspecific finding with a short differential.
Two secondary cases in experiment 1: Both cases contained evidence of appendicitis. One of the two also exhibited clinically relevant finding of free air, indicating a perforation of the viscus.

### 2.4 Case Selection: Experiment 2

Radiologists in experiment 2 viewed four CT cases. Neither of the experimental cases contained any critical findings. Both were abdominal CT scans.

### 2.5 Eye-Tracking Glasses and Analysis

Radiologists’ eye movements were tracked at a sampling rate of 60 Hz using mobile SMI eye-tracking glasses, which allow for the recording of both eyes with automatic parallax compensation with a spatial accuracy of ∼0.5  deg. The scene video was recorded at a resolution of 960×720 at 30  frames/s, with a field of view of 60 deg (horizontal) and 46 deg (vertical). Audio output from the eye-tracking glasses was examined when the radiologist spoke in response to telephone interruption in experiment 1 and in instances where dictation was unclear in both experiments. Event detection (segmenting samples into fixations and saccades) was performed offline with BeGaze software by SMI. Calibration took place prior to the experiment and involved asking participants to look at three different locations on the computer screen. The experimenter monitored eye-tracking software throughout both studies and recalibrated the eye-tracker between cases when necessary. One of 19 radiologists in experiment 1 had poor calibration, which rendered the eye-tracking data uninterpretable. Data from this radiologist were removed from subsequent analyses.

## 3 Results

### 3.1 Experiment 1

In order to evaluate the effect of interruption, we focused on the four experimental cases that each radiologist evaluated. On experimental cases that were interrupted, the telephone rang and when the radiologist answered a prerecorded message asked the radiologist to find and evaluate a second case, which we will refer to as the “secondary case.” This meant that the radiologist had to navigate to a different worklist in our software in order to find the second case. Once the secondary case was closed, the radiologist was able to immediately return to the experimental case without having to navigate back to the original worklist. Each radiologist was interrupted by a phone call twice during the ∼50  min experiment.

There are a number of different ways to calculate the cost of interruption. We adopted a conservative approach where the interruption was judged to officially begin at the moment when the secondary case was first opened. Our eye-tracking software allowed us to estimate this moment in time with 16-ms precision. The interruption was judged to have ended as soon as the secondary case was closed, thereby allowing the experimental case to be viewed again. A more liberal definition of interruption may have judged the interruption to have started at the moment the telephone rang, or when the phone was first answered. However, a number of our radiologists continued to examine the experimental cases while listening to the prerecorded message, making it difficult to be certain what they were attending during this time (experimental case or secondary case). Thus, to reduce ambiguity we focused on times where the radiologist appeared to be fully focused on the secondary task. To quantify the temporal cost of interruption, we subtracted time spent evaluating the interruption case. We then compared how much time was devoted to the interrupted and noninterrupted experimental cases during the first and second set of experimental trials.

We randomized the order and condition (interrupted or not interrupted) of the experimental cases across radiologists while holding the order of the filler cases constant. Thus, the first two experimental cases were the third and fourth cases viewed, and the second two experimental cases were the sixth and seventh cases viewed (see Fig. 1). We separated analyses of the first and second interruptions because we felt it was likely that the radiologist would treat the two events differently. The first interruption was innovative and may have been unexpected. Although we were careful to not inform the radiologists that the purpose of the study was to study interruptions, in order to ensure that they answered the phone, it was necessary to point out the phone and tell the radiologists to answer it if it rang during the study. Nonetheless, the second interruption was certainly more expected than the first interruption and we reasoned the response could differ accordingly. Thus, our design allowed us to pair each interrupted experimental case with an uninterrupted experimental case that occurred immediately after or before the comparison case: the third or fourth case for the first set, the seventh or eighth case for the second set.

Radiologists examined the first interrupted experimental case more than 2 min longer than the paired uninterrupted experimental case [interrupted cases: 11.1 min, uninterrupted: 8.8 min, t(18)=2.2, p<0.05, $\eta^{2}=0.23$]. However, the second set of interrupted experimental cases were evaluated for no longer than the paired uninterrupted experimental cases [interrupted: 7.0 min, uninterrupted: 7.1 min, t(18)=0.02, p=n.s., $\eta^{2}=0.00$]. Diagnostic accuracy followed a similar pattern, but the cost of interruption during the first set of experimental cases was not statistically significant in this sample [interrupted: 44.4% correct, uninterrupted: 61.1%, t(18)=0.9, p=n.s., $\eta^{2}=0.05$]. There was no effect of interruption on diagnostic accuracy on the second set of experimental trials [interrupted: 47.1% correct, uninterrupted: 41.1%, t(18)=0.34, p=n.s., $\eta^{2}=0.00$] see Fig. 3.

One might expect that the magnitude of the interruption cost was larger after the first interruption because more time was devoted to the first interruption. However, this was not the case. In fact, on average participants spent an equivalent amount of time on both interruptions [t(17)=1.09, p=0.29, $\eta^{2}=0.06$).

In order to better understand the cause of the time cost observed in response to the first interruption, we analyzed what proportion of time each radiologist spent on a set of critical areas of interest. To do so, we computed the aggregate dwell time of each fixation in each of these regions and divided by the cumulative dwell time for each case. We focused on three areas of interest: medical images, dictation screen, and other locations. Medical images were defined as any medical images viewed outside of the image navigation screen in the Philips Isite software. Radiologists spent a small amount of time examining the image navigation screen in order to choose what images to focus on, but the critical analysis of the images took place outside of this bookmark view of the images and was thus categorized as “medical image” dwell time. Time spent examining the navigation screen, other places such as the keyboard, or blank space between images was grouped together into the “other” category.

**Fig. 4:**
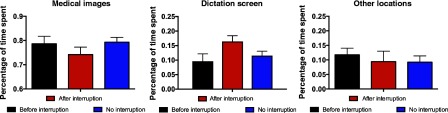
Percentage of time spent fixating on different areas of interest in experiment 2.

Figure 4 compares proportion of dwell time spent in these three interest areas for interruption cases prior to interruption, interruption cases after the interruption, and no interruption cases. To get a broad sense for whether the proportion of time spent in these three regions varied as function of the time window, we computed a repeated measures ANOVA with AOI and time window as factors. There was a significant effect of AOI [F(2,34)=142, p<0.001], but not time window [F(1,17)=0.31, p=0.58], and the factors interacted significantly [F(2,34)=4.3, p=0.02]. As a follow-up analysis, we compared the proportion of time spent on medical images and the dictation screen during the 30 s immediately after the interruption to trials where there was no interruption. The 30-s time window was based on previous work from our lab that has suggested that interruption effects are maximal during a short period of time immediately after interruption.^10^ Importantly, this prior work was conducted with naïve observers performing an analog to chest CT lung screening rather than radiologists examining real cases. We observed a significant interaction [F(1,17)=19.64, p=0.0004] between AOI (medical images or dictation screen) by time window (after interruption or no-interruption trial). This pattern was consistent during a 60-s time window after interruption as well.

#### 3.1.1 Sternal fracture case

**Fig. 5:**
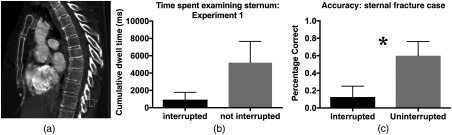
(a) An image from the sternal fracture case. (b) Cumulative dwell time of fixations near the sternum. (c) Accuracy of on the sternal fracture case as function of whether the case was interrupted or not.

Although we did not observe a reliable cost on diagnostic accuracy in the face of telephone interruption, in posthoc analysis we noticed a number of striking differences in how a single case was treated with and without interruption. While only one radiologist who was interrupted detected the unambiguous sternal fracture [see Fig. 5(a)], 60% of the radiologists who were not interrupted detected the same abnormality, a statistically reliable difference [t(16)=2.21, p=0.042, $\eta^{2}=0.23$]. To quantify how often and for how long the radiologists looked near the sternal fracture during this case, we reanalyzed the entire dataset. We found that time spent examining the region near the sternal fracture was dramatically different for radiologists who were interrupted (mean: 927 ms, range: 0 to 6870 ms, five out of eight never examined the region near the sternum), than those who were not interrupted (mean: 5205 ms, range: 0 to 23,150 ms, 2 out of 10 never fixated). While an unpaired t-test suggests that the difference in dwell time was not statistically significant [t(16)=1.49, p=0.16, $\eta^{2}=0.12$], the chance of a radiologist completing any fixations near the sternum was marginally statistically more likely on uninterrupted trials (80%) than interrupted trials [37.5%, t(16)=1.9, p=0.07, $\eta^{2}=0.19$]. Given the posthoc nature of these analyses and the small statistical effects observed, these results should be interpreted with caution. We will return to this issue in the discussion.

### 3.2 Experiment 2

**Fig. 6:**
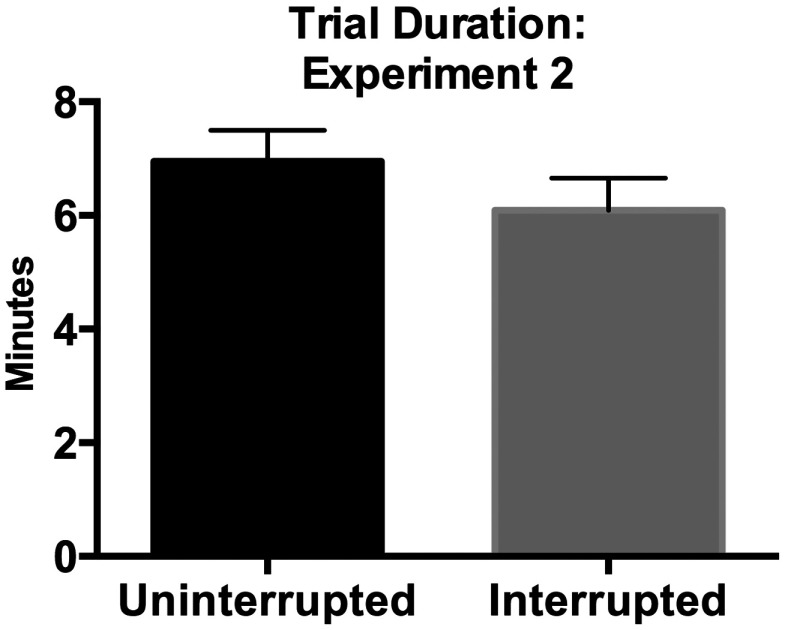
Trial duration for experimental trials in experiment 2.

In experiment 2, participants were interrupted once while working through a list of four cases. The interruption in this experiment occurred when the experimenter interrupted the radiologist during one of the two experimental cases and asked her to fill out a survey containing a series of demographic and radiological experience questions. See methods for more details. The experiment lasted ∼30  min. We defined the start of the interruption as the moment the participant first fixated on the questionnaire. We judged the interruption to be complete at the moment the radiologist fixated on any computer screen after filling out the form. Unlike experiment 1, we observed no time costs associated with interruption cases relative to matched uninterrupted cases [t(15)=−1.74, p=0.10, $\eta^{2}=0.17$, see Fig. 6].

In addition to a difference in the number of cases and the number of interruptions the participants experienced, the interruption in experiment 2 was different from that in experiment 1. Although we succeeded in creating a different interruption scenario that led to approximately the same amount of time away from the original case, there was a small but significant decrease in time devoted to the interruption in experiment 2 (1.6 min) relative to experiment 1 (2.2 min) [t(32)=2.98, p=0.006, $\eta^{2}=0.22$]. It is thus unclear whether the absence of an interruption cost in experiment 2 is due to less time spent on the interruption or the nature of the interruption itself. Future work will be necessary to clarify this issue.

**Fig. 7:**
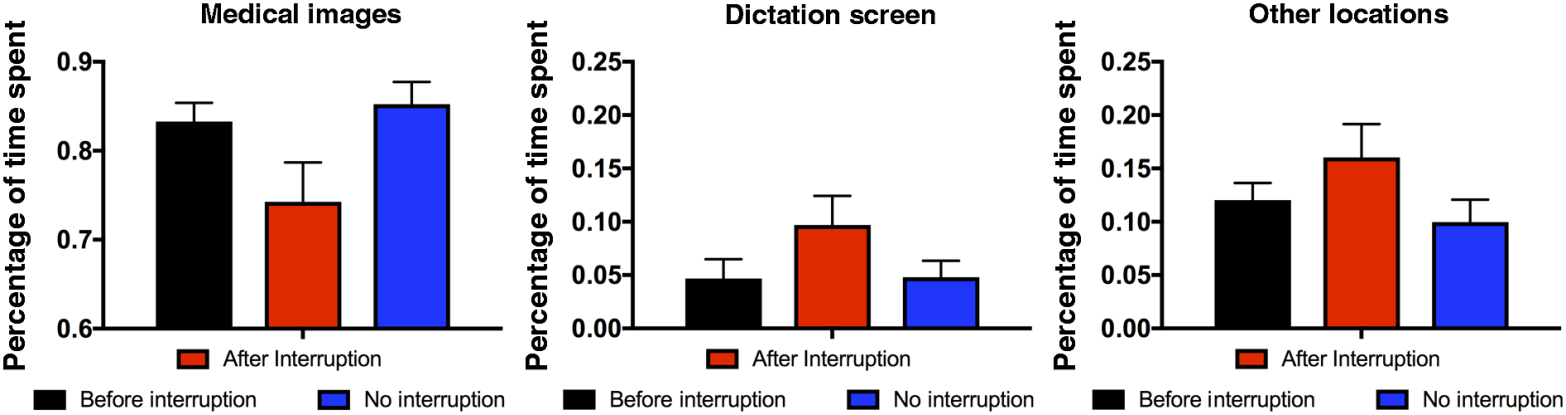
Percentage of time spent fixating on different areas of interest in experiment 1.

As in experiment 1, we compared proportion of dwell time spent in these three interest areas for interruption cases prior to interruption, interruption cases after the interruption, and no interruption cases (Fig. 7). We computed a repeated measures ANOVA with AOI and time window as factors. There was a significant effect of AOI [F(2,28)=234.1, p<0.001], but not time window [F(1,14)=0.85, p=0.37]. There was a trend toward a significant interaction between the two factors [F(2,28)=3.0, p=0.06]. As a follow-up analysis, we compared the proportion of time spent on medical images and the dictation screen during the 30 s immediately after the interruption to trials where there was no interruption. We observed a significant interaction [F(1,14)=5.68, p=0.03] between AOI (medical images or dictation screen) by time window (after interruption or no-interruption trial).

## 4 Discussion

In quantifying the adverse effects associated with interruption, the cognitive literature has focused on resumption lag (time to restart the primary task after completion of the interruption), interruption lag (time between the interruption alert and the start of the secondary task), total time on task (total time spent on the task minus time spent on the interruption), and task accuracy. Given our interest in reducing diagnostic errors and increasing patient throughput, we focused on total time on task and task accuracy.

When faced with resuming a case after being interrupted, the radiologist can either attempt to resume where they left off or start the case over from the beginning. We predicted that if they did start the case over, it would lead to substantial time costs, but this approach could also result in a benefit in diagnostic accuracy as it would provide a second chance to evaluate a portion of the patient’s case. Alternatively, if the radiologist attempts to resume where they had left off, they could miss parts of the patient’s case that they mistakenly thought they had already evaluated. We predict that this approach would lead to costs in diagnostic accuracy and no time cost. A third alternative is a mixture of the two extreme examples outlined above: the radiologist may attempt to recall where they left off and conservatively retrace their recent steps prior to the interruption to ensure that no areas are completely overlooked. We predict that this mixture model would lead to reliable time costs with very small costs on diagnostic accuracy that would only occur in the case of large memory errors with respect to what areas had and had not been evaluated.

While it is important to note that there are clear and important individual differences in approaches to addressing interruptions,^12^ our data appear to be most consistent with the mixture model above. In experiment 1, with a task relevant interruption that involved evaluating medical images, we observed a reliable time cost associated with the first interruption. There was no evidence of a time cost in the experiment 2, where the interruption was not relevant to the task and did not involve medical images. This is consistent with previous work from the cognitive literature: postinterruption resumption for a spatial task is markedly less accurate when the interruption task involves a spatial task.^13^

There was no evidence of a time cost in response to the second interruption in experiment 1. This is broadly consistent with findings in the cognitive interruption literature that have found that practice with a task leads to less disruption in response to interruptions.^14^^,^^15^ Nonetheless, we were surprised that there was no evidence of interruption time cost after just one prior interruption. Less surprising was the fact that no time cost was observed in experiment 2. Prior literature has found that interruption costs decrease when the secondary task is not related to the primary task. Our results appear consistent with this finding: the telephone interruption with a medical image perception secondary task was much more disruptive than the interpersonal interruption that required no image interpretation.

In contrast to the time cost associated with interruptions, the differences in which areas were focused upon during the period after interruption were reliably modulated in both experiments 1 and 2. During the postinterruption period, radiologists tended to spend more time looking at the dictation screen and less time looking at medical images. While the time costs associated with interruption were inconsistent across our experiments (though in directions that are consistent with previous interruption research as outlined above), these differences suggest that the interruption reduced the amount of time spent examining medical images even when an overall time cost was not observed. It is likely that the dictation screen serves as an external memory aid that helps the radiologist determine where they have and have not previously examined, which is predicted to be an effective strategy in the cognitive psychology literature.^7^ This suggests that radiologists are, on average, aware of memory limitations for where they have searched and use the dictation screen to address this issue. However, the fact that less time is being spent examining medical images after interruption without a concomitant increase in total time spent per case suggests that radiologists are spending less time examining medical images on interruption cases. In aggregate (for instance over the thousands of cases a given clinic examines each year), we expect this would lead to worse performance on interruption cases as a result. With just two interruptions in experiment 1 and no abnormalities to detect during the experimental case in experiment 2, the current study was not designed to detect what is likely a small effect on accuracy as a result of this change. However, we speculate that the decrease in time spent examining medical images observed in the current study may help explain the observed increase in discrepant calls during epochs of time with more phone calls in prior observational work.^3^ Given the enormous number of patients evaluated by radiologists, it is notable that even a small decrease in accuracy could influence the lives of thousands of patients per year.

One prediction from the decreased time attending to medical images is that overall coverage of crucial structures should be lower in interruption trials relative to uninterrupted trials. Coverage is a deceptively simple metric that is examined in many medical imaging perception studies that involve eye-tracking.^16^^–^^18^ The underlying assumption is that when the eye is at a discrete position in space, diagnostic information about the surrounding region with some radius (termed the useful field of view) is processed. Unfortunately, coverage is difficult to extract when using a mobile eye-tracking system as in the current work because the medical images and the eye-tracker do not automatically coregister. This means that any measures of coverage using traditional techniques would require mapping roughly 45 min of high-resolution video, resulting in ∼10,800 fixations per radiologist.

As a step in the direction of quantifying coverage, we examined dwell time in the region of a sternal fracture in experiment 1. The results of these analyses are consistent with the idea that interruptions lead to impaired examination of critical structures. Similar to findings in the cognitive psychology literature, interruptions may cause certain task steps (e.g., examining a particular structure) to be omitted. We found that a significantly higher proportion of radiologists detected the sternal fracture when they were not interrupted, and that the uninterrupted radiologists spent almost 5 s longer looking in the region of the sternal fracture. Notably, while both of these differences were nominally large, the effect on cumulative dwell time was not statistically reliable given the high variability across radiologists. As it stands, we see these results as promising initial findings that we hope to replicate and extend in future work.

An additional promising line for future research comes from discussions with the radiologist observers during study debriefing. A number of the radiologists noted that both interruptions employed in this study (phone call and interpersonal interruption) were benign relative to the disruptive interruptions they often face. In particular, they noted that it is extremely difficult to focus if interrupted more than once. In addition, they observed that interruptions that require leaving the reading room were particularly disruptive, often leading to the radiologist restarting the interrupted case. Of all the interruptions in the current study, we did not observe a single instance of a radiologist restarting a case after the interruption. Clearly, a stronger interruption manipulation is likely to have led to stronger observed costs.

In this context, it is impressive that we were able to observe reliable costs of interruption despite a design that allowed for just 1 to 2 relatively weak interruptions per radiologist. Overall, the effects were quite small: consistent effects of which areas were fixated on immediately after interruption, but a small effect on total time in some cases, and no effect on diagnostic accuracy. Given that radiologists now have to face interruptions quite frequently,^2^ perhaps they have devised effective methods for dealing with interruptions without the adverse effects typically observed in the cognitive interruption literature.^19^ While the cognitive interruption literature is focused on performance on a task that is new to the observer, in the current study we examined radiologist performance while performing a task familiar to all radiologists. Indeed, prior research suggests that expert surgeons demonstrate very little cost in response to a distracting interruption.^20^ It remains to be seen whether the actual costs of interruption in diagnostic radiology are actually quite small, or if they just appear small in response to one to two simple interruptions.

It is likely that case difficulty is an important factor in determining the magnitude of interruption costs. One would imagine that an interruption during a simple chest radiograph would be far less disruptive than complicated abdominal CT case. However, there is certainly a great deal of variability even within the category of what we consider complicated CT cases. From this perspective, the fact the current study examined the cost of interruption on just two (experiment 2) or four (experiment 1) cases is a significant limitation. Another approach to examine interruptions in a radiological setting would be to have a higher proportion of interrupted cases. This would have resulted in more interrupted cases and reduced the likelihood that our results are driven by aberrant responses to a particular case. However, in this first attempt to examine interruptions in a realistic radiology setting, we deliberately sought to create an experimental paradigm that was as close as possible to what a radiologist might experience during an overnight call shift. Although telephone interruptions are becoming quite common in radiological practice, it would be highly unusual to be interrupted on half of the cases during a ∼60  min stretch of time. Similarly, repeated interruptions may have encouraged the radiologist to adopt compensatory strategies in response to the frequent interruptions (e.g., be ready for an interruption near the beginning of each case).

An additional limitation is that the current sample of radiologists was skewed toward residents. It remains to be seen whether the effects observed in the current study would replicate in a sample of experienced attending radiologists. It is possible that attendings with more experience surmounting the difficulties associated with interruptions have developed effective methods to overcome problems associated with interruptions. Interestingly, we asked each radiologist if they had a specific strategy that they used when interrupted during actual practice. Although almost all radiologists reported some kind of strategy, there was a great deal of variability and no consensus, even within the cohort of participants from the same institution in experiment 1. We hope that future research can uncover a “best practices” approach to dealing with interruptions that could be passed along to future radiologists, but clearly more work needs to be done before attaining that goal.

We see this as preliminary research that is designed to help bridge the gap between the experimental research in the cognitive interruption literature and the more observational literature devoted to understanding the cost of interruption in radiology. The goal of this research is to finely understand the consequences associated with different types of interruptions. There is a wealth of research on factors that determine the severity of interruption costs in cognitive psychology literature that we are just beginning to apply specifically to diagnostic radiology. Ultimately, if we can identify factors that are particularly important in determining whether a given interruption will lead to adverse results, we may be able to use this research to design future reading room protocols that make these types of interruptions less frequent and/or less disruptive.
